# The inspired and the discouraged: School board service and differential effects on political ambition

**DOI:** 10.1371/journal.pone.0311054

**Published:** 2024-12-05

**Authors:** Kelly Bergstrand, Beth Anne Shelton, Rebecca E. Deen

**Affiliations:** 1 Department of Sociology and Anthropology, University of Texas at Arlington, Arlington, Texas, United States of America; 2 Department of Political Science, University of Texas at Arlington, Arlington, Texas, United States of America; PLoS ONE, UNITED STATES OF AMERICA

## Abstract

Given the national attention on Texas and its school board meetings, we ask—what are the effects of a contentious political atmosphere on desires to run for higher office? Further, how does this experience interact with individual-level traits to affect ambition? To investigate, we distributed a survey to elected school board trustees in Texas and analyzed quantitative and qualitative responses from 380 respondents. We find divergent paths, with some inspired and others deterred from future politics. Specifically, city residents were more affected, positively or negatively, than rural residents. Political newcomers, those feeling qualified, and Democrats were more likely to express higher ambition. In qualitative comments, the inspired wanted to make a difference, serve as a quality candidate, and represent others; the discouraged were upset by partisan politics, personal attacks, and constituents’ behaviors. Overall, these findings suggest that the larger political climate matters in shaping which individuals embrace or exit politics.

## Introduction

Since the start of the COVID-19 pandemic, school board meetings have been ground zero for fights over pandemic protocols. Previously routine, staid meetings turned raucous. Activists, community members, parents, school staff, teachers and students made impassioned and often contradictory pleas regarding masking, online instruction, and other strategies designed to mitigate the virus’s spread. At the same time, there has been a racial reckoning sparked by the murders of George Floyd, Breonna Taylor, Atatiana Jefferson and others, leading to arguments about how racial issues should be taught in school curricula, as well as demands for bans on books that discuss racial equity and racism. Furthermore, school boards have become a flash point when it comes to the presence of sex education or LGBTQ+ material in textbooks and classrooms, as well as rights for transgender children. While school board meetings have raged across the country, many of these battles played out vividly in Texas with school board meetings turning into sites for ideological fights, wrestling over microphones, physical confrontations, police involvement, and the need to shut down meetings [1]. Additionally, some school board trustees in Texas have personally experienced doxing, harassment, and even death threats [2].

What effect, if any, has this heightened politicization had on whether school board trustees choose to run for a higher office? Does the added attention and potentially rancorous atmosphere encourage members’ political ambition, or does the acrimony lead trustees away from higher office? Are all groups affected equally or are there differences in how geographic, demographic, political and civic-oriented traits shape political ambition in this new climate? This is a particularly important question since historically school board service can serve as a springboard for future political office. Furthermore, there may be demographic traits–such as race, gender, class, political orientation, and so on—that interact with the hyper-partisan atmosphere. Some may thrive in the new climate, while others may seek a quick exit. Understanding who is affected, and how, can shed light on not only who chooses to continue school board service, but also who is drawn more generally into higher office.

To answer our research questions, we surveyed all Texas school board trustees serving during the spring and summer of 2021 who represented public school districts. Texas is the second-most populous state in the U.S., with a large public school system serving over five million students housed in school districts of various sizes and attributes [3,4]. This helps provide quantitative and qualitative data from respondents with diverse traits serving in a variety of types of school districts. Not all Texas school districts are politically contentious, but given that some acrimonious school board meetings have made it into national or statewide news [1,2,5,6], we expect some of our respondents to reflect on how polarization has affected their experiences and ambition levels. We investigate the types of people whose ambition has been affected by their service on the school board and the ways that school board service has deterred people from or encouraged people to pursue higher office. We find three distinct groups: those whose ambition has been piqued, those whose service has dissuaded them from seeking higher office, and those for whom service has had no effect.

A majority of our respondents stated that school board service did not affect their political ambitions one way or another. Descriptively, this group differed demographically from the other two in that they tended to be older, live outside city or suburban districts, not have any children living at home, not be employed full-time, not participate in political groups, and to have served in elected positions before.

The respondents whose service on school boards affected their political ambition, either positively or negatively, were more likely to serve in urban districts. Respondents whose ambition was amplified, relative to those unaffected by school board service, tended to be Democrats, white, and political newcomers who felt they were qualified in a potential run for higher office. In qualitative comments, they expressed excitement about serving as a voice for others and being able to make a difference in their communities. By contrast, those whose service dissuaded them from future service were more concerned about negative campaigns than the group stating school board did not affect their ambition. In qualitative comments, these demotivated trustees also pointed to the toxicity of the current political environment, the personal attacks, and difficult constituents as reasons they are not interested in higher office.

Taken together, these findings highlight the need to better understand how a charged atmosphere of political and volunteer activities can interact with various demographic and civic traits to translate into individual-level ambition outcomes. This has important ramifications for who chooses to continue in, and who chooses to exit, work in civic and political arenas.

## Literature review

### Civic engagement and progressive ambition

To understand progressive ambition—the desire to seek higher office—one must first consider the process of becoming involved in civic life generally. School board members typically receive no compensation, making this potentially labor-intensive work a form of volunteering. The work of Verba, Schlozman and Brady (1995) is particularly helpful in understanding the processes at work to encourage and discourage civic and political engagement. In their Civic Volunteerism Model, there are three categories of prerequisites: skills, motivation, and recruitment [7]. In order to participate in civic and political life one must have the ability (to be able to communicate and to organize, for example). Volunteers are often distinguished by a number of human capital traits that can serve as resources to aid their work [8,9]. A person must also want to participate; something either innate in them or an experience might motivate them to want to get involved. A number of traits, including parental influences, socialization, concern for others and prosocial orientations, can contribute to this motivational aspect [9–11]. Often people are recruited into service, and in fact, some do not participate until someone invites them to become involved. Studies have found that being solicited substantially increases the probability of participating in politics, volunteering, and political activism [7,12–16].

Once elected, what leads people to pursue higher elected office? Scholars have been exploring this question for more than four decades [17–19]. In the past two decades, there has been robust work to understand the gender, race, and class dimensions of progressive ambition [20–28]. Another vein of inquiry has focused on the motivation piece of this puzzle, trying to understand the nuanced role that ambition plays [23,24,28].

### School boards as a springboard to higher office

The prospect of running an effective campaign can seem daunting. The structural barriers to standing for election to state legislature or the U.S. Congress are high, such as available time, money or access to financial donors, and, for some offices, being vetted by the party organizations. However, these obstacles are lower for local races and perhaps lowest for school board trustees. School board races are largely nonpartisan. The geographic area covered by a trustee is usually smaller than a legislative district. Often candidates are people who have developed a reputation in the community through volunteer work, professional networks, and other forms of civic engagement. If a person wants to get into electoral politics, the school board may be an attractive place to start. Having prior elected office on one’s resume also provides an important boost to being a viable candidate. One has experience campaigning, a deeper network of supporters and potential donors, and name recognition among voters–all of which are important to electoral success.

The potential for school boards to provide entry into politics can lead school boards to be a particularly relevant site for investigating candidate emergence and progressive ambition. A study of Texas school board trustees found that candidates for school boards regularly campaign on their civic and volunteer work, though with interesting gender differences [29]. Men came to office through traditional avenues of public life: membership in civic groups such as Rotary, on boards of non-profit organizations, and through volunteering (often in the school setting). While women had somewhat similar experiences, they were more likely to center their discussion on issues (school policies, curriculum, teacher/personnel issues) as motivating factors to running for office. Another study finds that successful female candidates almost uniformly had specific school related volunteer experience (like booster club or PTA board positions) whereas male candidates did not [30].

Additionally, demographic differences, especially gender, can be important factors in how school board experiences translate into progressive ambition. In a study of school board trustees in Pennsylvania, the author found men and women differed most on the factors that influence their progressive ambition. Among men, education, efficacy, qualifications, and personal networks (family, friends, co-workers, spouse) feature prominently [28]. For women, education is not significant, but both personal and political networks (elected officials, activists, and party elites) are important predictors of progressive ambition. Interestingly, she also finds that the levels of support in both personal and political networks are lower for women than for men. Race can also matter, with minority candidates less likely to win school board election campaigns; one study in Ontario found that while racial minorities made up about 20% of candidates, only 6% became elected trustees [31].

### Pandemic politics and hyperpolarization

While we understand quite a bit about the gendered nature of political ambition, and a bit about how that plays out among elected school board members, we are just learning about the effects of the pandemic on politics. Our understanding of school boards as entry sites for progressive ambition may need to change if the climate around school board meetings becomes one fraught with sanctions and deterrents. School board elections are generally not partisan, and early studies found few demands of constituents beyond taxes to filter to trustees [32]. Partisan discourse is increasing, however, as national trends are felt locally [33,34]. Conflict among trustees might also be related to low school performance [35]. Recent reports and scholarship have documented that partisanship affected views on going back to school online or in person in fall 2020 [36,37]. Additionally, political affiliation was highly correlated with views about the desirability of children returning to in-person learning [34] and with states’ choices about lockdowns [38]. By the fall of 2020, most states left the decision about whether to return to in-person learning up to the localities, and there is evidence from Michigan that these local decisions were colored by partisanship [39]. Republicans were much more in favor of children physically returning to class.

School board meetings have also turned into sites of activism. While social movements can and do focus on policy changes, many also focus on cultural elements, such as winning hearts and minds, affecting public opinion, or changing everyday lifestyles and behaviors [40,41]. Activists have specifically focused attention on school board meetings as a way to articulate and contest various visions for what values and types of knowledge should be encouraged or banned. This has turned routine meetings into sites of contention and has raised attention and pressure on school board members, whose votes are not just a matter of crafting school policies, but seen by some groups as a referendum on the types of beliefs and attitudes that should be promulgated in society. This new environment has benefits in terms of elevating the perceived impact of elected school board trustees’ actions (potentially making the work feel more influential), but can also come with added scrutiny and pressure (making the work more unpleasant or stressful). Despite nationwide attention on educational policies as a reflection of cultural priorities and activism, there is surprisingly little sociological work on school boards themselves.

To help understand any new effects on progressive ambition that might emerge from serving on school boards in a hyperpolarized atmosphere, we look at the case study of Texas, which is known for its school board battles. How might progressive ambition be affected in a potentially contentious environment? Are all individuals affected in the same way by their school board service, or do demographic, political, and geographic traits shape differences in whether people are inspired or dissuaded from pursuing higher office?

## Methods, measures, and analysis

### Case study

To investigate our research questions, we selected the case study of Texas, which has made both statewide and national news due to contentious political events at the school governance and school board levels [1,2,5,6]. It is important to note that not all Texas school boards experience controversy surrounding their decisions. However, overall, the state does tend to be a flash point for issues with known political divisions that arouse strong opinions and emotions. As an example, between 2021–2022, Texas removed more books from its school library shelves than any other state in the U.S., with most of the removed books discussing issues such as race, racism, abortion and LGBTQ issues [6]. Debates about book bans, as well as other topics such as covid-era policies, school shootings and safety, sex education, sexual orientation, and transgender students’ rights can then spill into local school board meetings [1,2,5,6]. Texas is also a sizable case study, with over 30 million residents, and high levels of racial and ethnic diversity, particularly in regard to Latino Texans [4]. The state has over five million public school students and a mix of school districts of various sizes in urban, rural, and suburban areas [3]. Thus, this case study provides us with a large and diverse set of respondents for both our quantitative and qualitative data collection. In Texas, the boards of independent school districts are elected positions charged with governing and overseeing the education in their local school district. In general, school boards in the United States have a fair amount of power and autonomy over local school governance [42]. Thus, the conclusions from this study are most applicable to countries in which school boards are elected positions. It is less applicable to situations with appointed school board positions or non-democratic countries that tightly control and regulate their educational systems to ensure pro-state educational stances [43]. Further, since our key outcome is interest in running for political office, it is most applicable to democratic countries that have elected positions.

### Participants

To investigate our research questions, we sent an online survey to all individuals with an available email address who were serving as an elected school board trustee for Texas public school districts in 2020–2021; this did not include members who were elected in the fall of 2021. This project was approved by the University of Texas at Arlington’s Institutional Review Board (Protocol 2021–0660), no minors were included in the study, written informed consent was obtained, and recruitment occurred between August 3, 2021 and November 5, 2021. Our response rate was 12.8%. We then dropped any participants who did not complete the survey, resulting in 380 total respondents. We provide data on the traits of participants in Table 1. Overall, our respondents were majority male (60%), white (75%), married (90%), employed (74%), and educated with a college degree or higher (79%). About half had children living at home (54%), and about half were 55 or older (49%). Most had household incomes higher than $100,000 (74%). Of the respondents, 41% attended meetings of political groups and 40% were on school boards in large suburbs or cities. About half are Republican (53%), while the rest are split between Democrats (20%), Independents (24%), and Other (3%). We also collected data on district type and gender to further assess how well our sample mirrors the population of trustees surveyed. The representativeness of our sample in terms of district type is quite good. For all but three district types our sample is within 2–3 percentage points of the representation of district types in the population surveyed. We have a slight overrepresentation of trustees from rural distant districts (less than 4 percentage points), somewhat larger overrepresentation of trustees from large suburban districts (almost 8 percentage point difference) and an underrepresentation of trustees from distant towns (just over 6 percentage points). The only other demographic data we have for both the population and the sample is gender. The population of trustees surveyed is 54.7% women and our sample is 40.2% women. Respondents from larger school districts are more representative of the gender composition of those districts than respondents from smaller districts.

**Table 1:** Descriptive statistics for each group, mean (SD) or %.

	All Groups	More Likely	Less Likely	Not Affected
Income over 100K	74.49%	74.36%	85.45%*	72.12%
Male	59.74%	64.29%	51.61%	61.14%
Have Children	54.18%	61.45%	61.29%	49.77%*
55 or Older	49.07%	34.94%*	35.48%*	57.71%*
Democrat	20.42%	32.14%*	14.52%	17.70%
White	75.48%	70.37%	82.46%	75.89%
BA Degree or Higher	79.47%	80.95%	88.71%*	76.42%
Employed	74.21%	88.10%*	75.81%	68.56%*
Married	90.08%	90.48%	90.00%	89.73%
Political Group	40.80%	52.50%*	41.94%	36.45%*
City District	40.00%	47.62%	53.23%*	33.19%*
Prior Elected Office	12.40%	5.95%*	8.06%	16.23%*
Feel Unqualified	2.37 (1.23)	1.88 (.870)*	2.51 (1.39)	2.49 (1.20)*
Concern Negative Campaign	4.45 (1.92)	4.26 (1.88)	5.33 (1.99)*	4.29 (1.87)*
COVID Lower Ambition	2.72 (1.69)	2.55 (1.68)	3.29 (2.03)*	2.62 (1.56)
*N*	380	84	62	229

Notes: The sample size given for groups is the total sample size; for each factor sample size will depend on missing data. Asterisks indicate that the difference in the proportion (or mean) is significant at p < .05 for the specified group compared to the other two groups combined.

### Measures

#### Dependent variables

The primary outcomes of interest focus on the question: Has serving on the school board changed whether you would consider running for higher office? The answer choices were: No, it has not had an effect; Yes, it has made it more likely; or Yes, it has made it less likely. Of the 375 respondents who answered this question, 229 stated it had no effect, 84 stated it made it more likely, and 62 said it had made it less likely. If respondents answered yes, they were then routed to a follow-up open-ended question that asked them to explain why it had made them more [or less] likely to run for office. We also analyze a question that asks trustees how likely it is (1 = Extremely likely, 7 = Extremely unlikely) that they would run for higher office in the future, listing these positions: City Council, Mayor, State Representative, State Senator, Judge or District Attorney, U.S. House, U.S. Senate, Governor (or other statewide office), and President.

#### Independent variables

We include several measures that could affect the school board experience or shape progressive ambition. Using categories from the National Center for Educational Statistics, we control for where the school district is located: City district (1 = school district is in a large suburb or city; 0 = school district is in a midsized or small suburb, town, or rural area). We include a question asking respondents, regardless of their interest in running for higher office, overall how qualified they felt they would be for higher office (1 = Extremely qualified, 7 = Extremely unqualified). Additionally, we ask respondents whether they agreed or disagreed that they were concerned about a negative campaign when running for higher office (1 = Strongly disagree, 7 = Strongly agree). We also included a measure of how frequently respondents attended meetings of political groups (recoded to 0 = never attended meetings, 1 = attended meetings once a year or more) and whether they had served in an elected position prior to running for school board (1 = Yes, 0 = No). Given the unique pressures of the COVID era, we included a control for whether the respondent agreed or disagreed with the statement that COVID (specifically) lowered their ambition to run for higher office (1 = Strongly disagree to 7 = Strongly agree).

#### Demographics and additional controls

We included basic demographic characteristics in our analysis. However, because our sample tended to lack variation, many of these were recoded into dichotomous variables. For example, only one respondent was in the lowest income bracket of less than $20,000, only six had a trade or vocational education, only six were single/never married, and just three were under the age of 34. The demographic variables included income (11 point scale recoded to 1 = $100,000 or higher, 0 = less than $100,000); sex (0 = Female, 1 = Male); age (7 point scale recoded to 1 = 55 and over, 0 = younger than 55); education (8 point scale recoded to 1 = Bachelor’s degree or higher, 0 = less than a Bachelor’s degree); Race (recoded to 1 = White, 0 = all other racial/ethnic categories); marital status (recoded to 1 = Married/Partnered, 0 = all other categories); children living at home (1 = Yes, 0 = No); Democrat (recoded to 1 = Democrat, 0 = Independent/Republican/Something else); and Employed (recoded to 1 = full-time, part-time, self-employment or military, 0 = unemployed, student, homemaker or retired).

### Analysis

The analysis we present here unfolds in several stages. First, we present information about each of the three groups of school board trustees. Further, we provide information on the nature of progressive ambition by looking at what positions the various groups might run for in the future. Next, we conduct a multinomial logistic regression on interest in running for higher office after serving on the school board, comparing those who state they were not affected to those more likely to seek higher office, as well as those less likely to seek higher office. We also plot the predicted probabilities of falling into the more ambitious, less ambitious, or unaffected groups. We used Stata 15 for our statistical analysis and listwise deletion for missing data. We also conduct a qualitative analysis on the 147 open-ended responses to two questions: explaining why service on school board made it more or less likely to run for office; or what else respondents would like to share. This allows us to take a deeper dive into the reasons, motivations, and mechanisms driving how school board service has affected respondents’ political ambition.

Responses to our open-ended questions were coded independently by two members of the research team employing grounded theory methodology [44–46]. Each author coded responses to the open-ended questions line-by-line and tagged responses with codes reflecting the sentiments expressed. After the initial creation of codes, the two authors who coded independently compared assessments, resolved any differences in coding and created a uniform set of codes that reflected their analyses.

## Results

### Demographic differences across levels of progressive ambition

The descriptive results provide demographic portraits of these three groups; the percentages discussed below are significantly different for each group (relative to the proportion of the two other groups combined) using a two-sample test of proportions. Additionally, Table 1 presents a breakdown of demographic differences for each of these three groups.

Respondents unaffected by their time on the school board are significantly more likely than the other groups to be to be older and to have previously served in an elected position. Specifically, 58% are 55 or older (while the other two groups only have 35% over 55). The unaffected group has the lowest rates of being employed, with almost a third being retired (28%) and are less likely to have children living at home. The not affected group is also more likely to have served in elected positions before (16%), twice the rate of the other two groups. They also have the lowest rates of being a member of a political group. They are also more likely to be embedded in small towns and rural areas, with the lowest percentage in city districts (at 33%) compared to those more (48%) or less (53%) likely to run.

Respondents more likely to run for higher office after their time on the school board are significantly less likely to have previously served in elected positions before relative to the other groups. However, they are more likely to be in political groups (53%). They are also significantly more likely to be Democrats (32%) with over twice the rate of those less likely to run for office. They are employed at higher rates (88%). This group also had the lowest mean of feeling unqualified (1.88) compared to the other two groups (≈2.5), showing their relatively high levels of confidence.

Like those respondents whose progressive ambition was positively affected, respondents less likely to run for higher office after their time on the school board are also more likely to live in a city (53%). Thus, those in cities were far more likely to have their ambition levels affected by school board service (positively or negatively) than trustees located in smaller towns or rural areas. Those stating they were less interested in running for future office also tended to be the most privileged of the three groups, with larger percentages having high income (over $100,000) (85%) or a college degree or higher (89%) than those in the unaffected or energized to run for office groups. This group had the highest average concerns about negative campaigns and was the most likely of the three groups to agree that COVID lowered their ambition.

### Do school board trustees have progressive ambition?

Next, we examine whether school board trustees in Texas exhibit progressive ambition. We asked trustees the likelihood on a seven-point scale of their running for a series of higher offices: City Council, Mayor, State Representative, State Senator, Judge or District Attorney, U.S. House, U.S. Senate, Governor (or other statewide office), and President (See Table 2).

**Table 2:** Progressive ambition by political office.

	Extremely Likely	Somewhat Likely	Likely	Neither	Unlikely	Somewhat Unlikely	Extremely Unlikely
City Council	12(3.2%)	12(3.2%)	38(10%)	36(9.5%)	23(6.1%)	70(17.5%)	186(49.3%)
Mayor	11(2.9%)	9(2.4%)	18(4.8%)	33(8.8%)	19(5.1%)	62(16.5%)	222(59.4%)
State Rep.	10(2.7%)	14(3.7%)	40(10.6%)	37(9.8%)	16(4.2%)	61(16.1%)	198(52.7%)
State Senator	6(1.6%)	8(2.1%)	34(9.0%)	34(9.0%)	14(3.7%)	66(17.5%)	214(56.9%)
U.S. House	5(1.3%)	6(1.6%)	22(5.8%)	35(9.3%)	16(4.2%)	67(17.8%)	224(59.7%)
U.S. Senate	6(1.6%)	4(1.1%)	22(5.8%)	28(7.4%)	10(2.7%)	67(17.8%)	238(63.5%)
Governor	3(0.8%)	2(0.5%)	8(2.1%)	26(6.9%)	9(2.4%)	61(16.3%)	264(70.8%)
President	2(0.5%)	2(0.5%)	2(0.5%)	24(6.4%)	4(1.1%)	44(11.2%)	297(79.2%)
Judge or D.A.	3(0.8%)	3(0.8%)	10(2.7%)	26(6.9%)	7(1.9%)	47(12.5%)	278(74.3%)

Overall, most trustees are not likely to run for higher office. The modal category across all offices is “extremely unlikely.” Also, as one would expect, the likelihood of running for a future office is greatest at the municipal or state representative level. For example, the top three positions that respondents replied they were extremely, somewhat, or likely to run for were state representative (17%), city council (16%), and state senator (13%).

We then examined these trends separately for those trustees for whom their school board service increased their progressive ambition: those that responded that it made them more likely to run for higher office (See Table 3). There are clear differences in this subgroup of trustees. If we compare across offices in all versions of likely to run, we see that while city council remains popular (39.7%), state representative is an office for which these progressively ambitious trustees are interested in running (43.2%). State senator is more popular (34.5%) than mayor (24.4%). In terms of affecting policy changes, this distribution of interest shows a political sophistication and knowledge of policy making (legislative bodies) versus policy implementation (mayor). What about for those trustees whose service has discouraged them from seeking higher office? As one might expect, those who reported that their service decreased their ambition were also not likely to report being interested in any of the offices on which we measured ambition.

**Table 3:** Progressive ambition for respondents with increased ambition, by office.

	Extremely Likely	Somewhat Likely	Likely	Neither	Unlikely	Somewhat Unlikely	Extremely Unlikely
City Council	7(8.4%)	7(8.4%)	19(22.9%)	11(13.3%)	5(6.0%)	10(12.1%)	24(28.9%)
Mayor	5(6.1%)	5(6.1%)	10(12.2%)	13(15.9%)	8(9.8%)	9(11.0%)	32(39.0%)
State Rep.	4(4.8%)	11(13.4%)	21(25.0%)	17(20.2%)	7(8.3%)	7(8.3%)	17(20.2%)
State Senator	2(2.4%)	7(8.3%)	20(23.8%)	13(15.5%)	7(8.3%)	10(11.9%)	25(29.8%)
U.S. House	2(2.4%)	5(6.0%)	14(16.9%)	14(16.9%)	9(10.8%)	12(14.5%)	27(32.5%)
U.S. Senate	2(2.4%)	3(3.6%)	14(16.9%)	13(15.7%)	5(6.2%)	13(15.7%)	33(36.8%)
Governor	2(2.4%)	2(2.4%)	3(3.6%)	10(12.1%)	7(8.4%)	16(19.3%)	43(51.8%)
President	1(1.2%)	2(2.4%)	1(1.2%)	8(9.6%)	0	12(14.5%)	59(71.1%)
Judge or D.A.	1(1.2%)	1(1.2%)	2(2.4%)	12(14.5%)	2(2.4%)	9(10.8%)	56(67.5%)

### Predicting increased ambition or decreased ambition

While the descriptive statistics indicate substantive differences in the demographic make-up and progressive ambitions of these three groups, multinomial logistic regression can assess how specific traits predict our outcomes. To understand how people might be affected by their school board service in different ways, we ran models to compare those for whom service increased their ambition as well as those for whom being a trustee decreased their progressive ambition, to those who reported it had no effect.

#### Increased ambition

**Fig 1:**
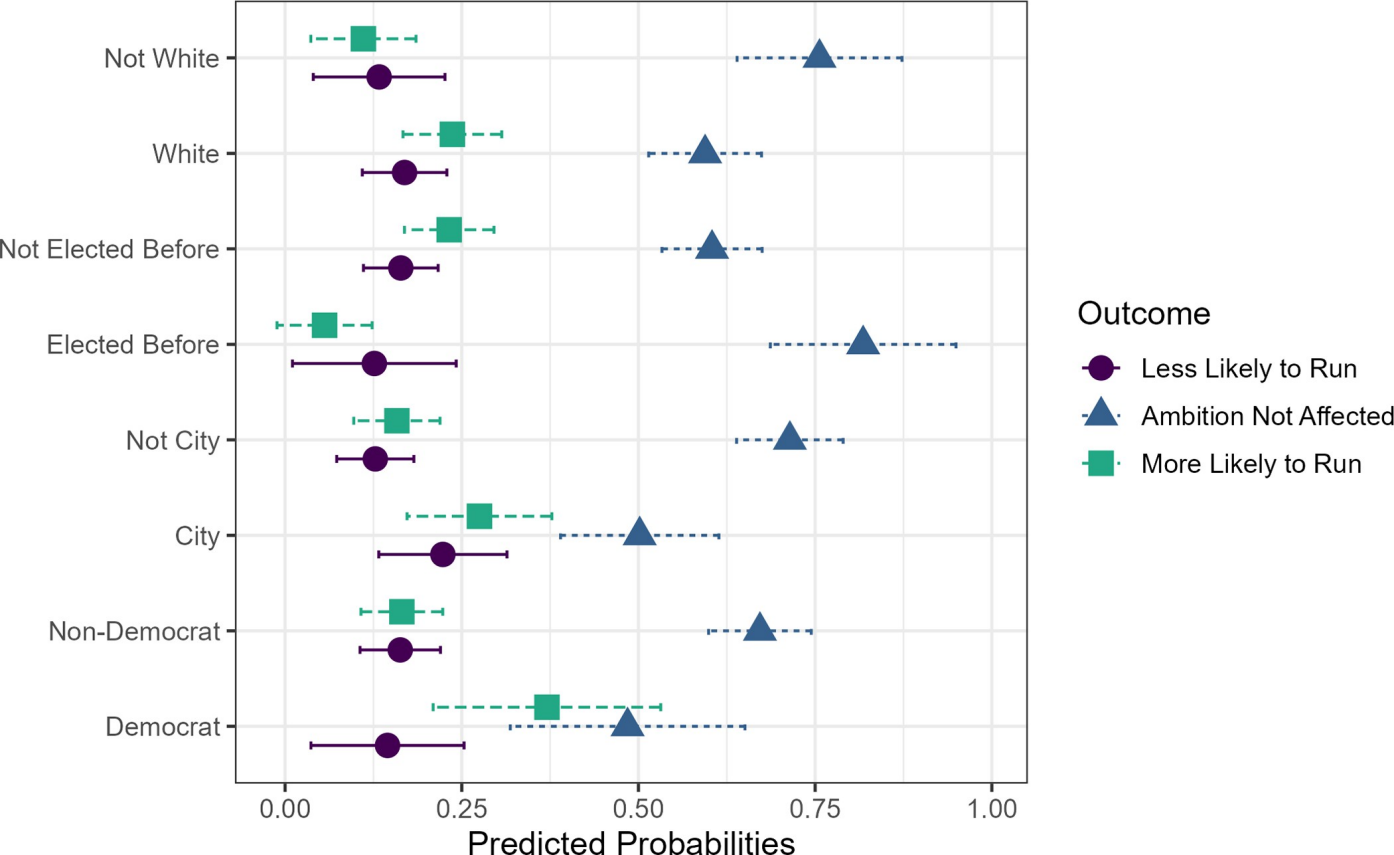
Predicted probabilities of ambition levels with CI (95%).

Both the quantitative and qualitative analyses reveal a distinct group of trustees whose progressive ambition was heightened by their service. When we model the likelihood of being in this group relative to the unaffected, we learn that one key factor is being a Democrat (b = 1.13, *p* = .0096, see Table 4). The predicted probability of being in the increased ambition group increases from 17% for non-Democrats to 37% for Democrats (see Fig 1). We also see significant positive effects on ambition for white respondents (b = 1.00, *p* = .02) and for those who live in school districts in cities or large suburbs (b = .91, *p* = .0099). One possibility is that school board meetings in larger cities could have attracted more attention (including from activists), making them more public battlegrounds than their rural counterparts. This could have had a galvanizing effect, relative to the comparison group of being unaffected by school board service. The predicted probabilities also reveal higher rates of progressive ambition for those in cities or large suburbs (28%) compared to those in smaller towns or rural areas (16%). Two traits decreased the probability of being in the more likely to run for higher office. Unsurprisingly, feeling unqualified for higher office had a negative effect on progressive ambition (b = -.777, *p* < .001) but having more experience—serving in an elected position prior to running for school board—also had a negative effect (b = -1.73, *p* = .0095). The predicted probability of being in the more likely to run group goes from 6% for those previously elected to 23% for those never before elected to a position. This could be because more experienced political officials saw the effects of the COVID era on the school board as “business as usual” and were more likely to fall into the unaffected group. But it also suggests that those most energized were political newcomers.

**Table 4:** Multinomial logistic regression predicting school board effect on ambition.

Model 1: Less Likely Model 2: More Likely to Run for Higher Office to Run for Higher Office				
High Income	0.34	(0.53)	-0.71^+^	(0.42)
Male	-0.02	(0.41)	0.10	(0.39)
Children	0.22	(0.47)	0.03	(0.42)
Democrat	0.21	(0.54)	1.13**	(0.44)
Over 55	-0.35	(0.46)	-0.80^+^	(0.44)
White	0.48	(0.49)	1.00*	(0.43)
BA Degree or More	0.48	(0.51)	-0.24	(0.42)
Employed Full-Time	0.22	(0.50)	0.62	(0.50)
Married	0.35	(0.70)	0.19	(0.56)
Political Group	0.30	(0.38)	0.47	(0.34)
City District	0.91*	(0.38)	0.91**	(0.35)
Previous Elected Position	-0.56	(0.57)	-1.73**	(0.67)
Feel Unqualified	-0.06	(0.15)	-0.78***	(0.21)
Concern Negative Campaign	0.20*	(0.10)	-0.01	(0.09)
COVID Lower Ambition	0.17^+^	(0.10)	0.03	(0.10)
Constant	-4.43***	(1.30)	-0.32	(1.17)
*N* 289 McFadden’s Pseudo *R*^2^ .152				
		

Notes: Regression coefficients presented; standard errors in parentheses. Reference group consist of those stating that serving on the school board had no effect on running for higher office.

^+^ *p* < .10

* *p* < .05

** *p* < .01

*** *p* < .001.

Now that we have a sense of the kinds of trustees who are in this group, how did they explain their increased ambition? While the survey data provide a snapshot of overall trends, the qualitative comments provide an opportunity to take a deeper dive and look at the content of their ambition to serve in higher office.

Among respondents for whom service on the school board increased their interest in running for political office, one common reason was a desire to make a difference. As school boards become a battleground for larger cultural issues and gain public scrutiny, service could be viewed as an opportunity rather than a burden. While having people fighting in school board meetings is far from ideal, it does indicate that people feel deeply about issues and that there are real consequences for students at stake. For these respondents, rather than scare them off, the school board experiences inspired them to do more. One respondent summarizes, “It’s made me realize that one person CAN make a difference, and that if I have an issue with something, there are likely others that feel the same as I do.” We see this language mirrored in several comments, such as those stating, “I see what a change I can make” and “I see how to make a difference.” This could then translate into progressive ambition, where they now have excitement for making even more of an impact in higher office. Many saw a need in politics for their abilities and believed that achieving higher office would allow them to be even more effective. For example, one respondent noted that “I believe some of the things I would like to accomplish require a higher level of governance to attain.” In another example, when asked to explain his progressive ambition, a school board trustee states: “Because the work that needs to be done to have a true impact on students is at a state level.” Thus, progressive ambition is fostered, in part, because respondents see expanded possibilities through higher office for implementing the changes they seek.

For those more likely to run for higher office, another related theme was the need for quality elected officials in it for the right reasons. Not all trustees were deflated by the negative atmosphere; some presented the counterargument that they needed to work in elected office to contribute in positive ways when others did not. They expressed the desire to continue in politics to serve as a counter presence to others, such as a respondent who states, “I believe there needs to be a voice for those who can see the best in humanity and not pander to fear and incite hatred.” Another trustee echoes this sentiment with, “We need more people with a heart of service and less politics.” The emphasis here is on providing a different perspective or approach than the negativity or hyperpolarization seen in some school board meetings. Similarly, respondents sometimes framed their progressive ambition in terms of feeling that there was a responsibility to have more qualified, competent individuals in office. For example, one respondent states, “The need for good governance in all elected posts is much more apparent than before.” Here, the focus includes the need to run for office, not just to provide skills and honest intentions, but to prevent other less qualified or ill-intentioned candidates from achieving office and power.

Finally, among this inspired group, there were a few who felt responsible for serving as a voice for others. For example, one woman states, “It has helped to be a voice for those that feel they have none.” Another school board member goes into further detail about this sentiment and highlights the importance of speaking for those who might be underrepresented. She states:

I represent a group of the population that has been historically marginalized when it comes to having a seat at the table. Seeing the value of my representation has motivated me and opened me up to the duty I have to sit at other tables where decisions are being made that affect everyone, including segments of the community that are often times overlooked.

For these individuals, being in a position of power allowed them to bring in underrepresented perspectives and to help counter exclusion. Ultimately, the respondents energized for seeking higher office saw the potential to create change, ensure the right people were in the job, and act on behalf of others. The school board climate was an inspiration rather than a deterrent.

#### Decreased ambition

The story is different for those school board trustees whose service led them to reject running for higher office. Interestingly, being in a city district also increased the probability of being less likely to run for higher office, compared to being unaffected (b = .91, *p* = .015). Additionally, concerns about a negative campaign (b = .20, *p* = .048) increased the likelihood of being less enthusiastic about running for higher office (see Table 4). The predicted probability for being in the less likely to run for office group goes from 13% for non-city districts to 22% for those in city districts. School board meetings in cities may have attracted extra attention as some conservative groups advocate and train protestors to travel to school board meetings, usually in cities [47].

These findings are confirmed in our qualitative analysis as these trustees tell us very clearly that they are turned off by the toxicity, “dirty politics,” and demanding people (among other things) they encounter as school board trustees. While the more likely to run group is invigorated and ready to make a difference, the less likely to run group is ready to retreat. Thirty-five percent of those who were less likely to want to seek higher office after serving on the school board cite the polarized or negative climate as the reason. Another fifteen percent cite demands made by constituents or problematic people as the reason. For this group of trustees, the current climate, in one form or another, is depressing ambition. One trustee puts this bluntly: “The current environment is toxic.”

Of those identifying the political atmosphere as a reason not to seek higher office, many brought up the polarizing or partisan environment. For example, a respondent states that “a nonpartisan role has been made not only partisan, but ugly.” Another school board member cites the “partisanship in all levels of political office” as a barrier to running for higher office. This political climate led many to feel that they could not complete their goals or enjoy their work on the school board. One trustee discussed how he unsuccessfully tried to reduce the amount of partisan politics on the school board, with the end conclusion that: “The election process and the election of politically motivated and poorly qualified people make it a challenge to focus on the primary work of the board. I realize that I do not like partisan politics.” While the group more likely to run for office sees political or unqualified candidates as a reason to run themselves, the less likely group emphasizes how these individuals make their work more difficult. This was a common sentiment among respondents; that partisanship and polarization in the political environment made the school board experience particularly unpleasant. One trustee explains this more fully with her assessment:

Local (and national) harassment of volunteer (unpaid) school board members is a shame. What is happening in today’s society will discourage normal citizens and parents who are honestly in the middle and have no party allegiance to stop volunteering, and this will negatively impact our entire public school system. I will not run again, nor would I make a fantastic candidate feel guilty about not wanting to serve in this climate. It is a sad reflection on the state of our country.

The challenges of both the pandemic and political polarization on school boards has made national headlines, so it is unsurprising that respondents seeking to exit politics or curtail their ambitions emphasize these problems.

Another prominent theme in the qualitative comments was unhappiness with the negative actions directed at the trustees and their families. Some were upset at personal attacks, such as one man who comments, “The things public elected officials have to go through and get accused of or called is terrible. It is sad that people who simply want to serve are treated so poorly.” Another trustee echoes this feeling with, “In the current political climate I have been unfairly and untruthfully maligned and received irreparable damage to my reputation.” And another respondent laments, “I never thought I would experience character assassination in a school board election.” Other school board trustees pointed to stresses placed on their spouses and even their children in the school system. They missed being able to go out in public and engage in community activities without having to hear complaints from community members. The result was that many expressed a desire to leave their current role on the school board, much less explore a career in other elected positions. As one man put it, “The constant need to address individual concerns that are often emotion based and meanness would likely intensify the higher up. No thanks.”

Finally, respondents identified constituents’ unreasonable behavior and demands as a reason not to seek higher office. These complaints range from the demands of constituents (e.g., “the constant need to address individual needs”) to the negative behavior of constituents (e.g., “dealing with highly polarized public opinions that don’t leave room for reasonable compromises”). One trustee put it succinctly:

I have so much to offer as a caring, thoughtful, inclusive, and highly educated (college professor with PhD) official, but these past 2 years have exposed the worst of people. I began my first term willing to miss my kids’ basketball games for my elected school board duties. Now, after having so many situations of people screaming and be[ing] completely uncivilized in meetings, I’d prefer to leave my board seat to someone who isn’t missing her son’s piano recital for that treatment. Life is too short. I won’t run again. This is why good people leave.

## Discussion

We find important demographic, political, and geographic differences between those whose ambition was affected by their service and those for whom it made no difference. Our three prongs of analysis–descriptive, regression analysis, and qualitative comments—reveal three distinct portraits for these three groups.

Respondents more likely to run for higher office after their time on the school board were a confident, active, and galvanized group. Descriptive results show that they tended to have higher rates of belonging to political groups, be younger, identify as Democrats, be employed, feel qualified to run for higher office, and be political newcomers. The multinomial logistic regression analysis offered further clarity on these descriptive trends–showing that being Democrat and white increased the probability that school board service motivated school board trustees to seek higher office, relative to those unaffected. Both feeling qualified to run for higher office and not serving in elected office previously increased the odds of higher political ambition. Living in city or suburban districts, where many of the high-profile, publicized school board meetings are held, also affected ambition, both positively and negatively. Qualitative comments reveal that this more likely to run group are energized to be a voice for others and to make a difference. Their time as a school board trustee led to themes such as respondents believing there was a need for the right people in elected positions (e.g., competent and focused on service), that they saw what a difference they could make, and that they believed that more political power could amplify their existing voice or bring about more changes. Thus, for this group, school board service was mobilizing and empowering.

Respondents less likely to run for higher office after their time on the school board expressed disdain for the hyper-partisan environment and fears of the negative aspects of campaigning. Descriptive results show that, in many ways, this group had the most resources of the three. They tended to have higher incomes and higher levels of education than the other groups. They also tended to be younger than the unaffected group. They were more likely to agree that COVID lowered their ambition to run for higher office. The multinomial logistic regression results reveal that concern about the negative aspects of trying to campaign for higher office was an important factor in having decreased ambition, compared to the unaffected group. Like the more ambitious group, they also lived in city or large suburban districts where much of the activism and controversy were centered, but unlike their galvanized peers, this extra attention and rancor was not a source of inspiration, but a deterrent to their political ambition. Qualitative comments reveal that trustees less likely to run for higher office were tired of the toxic environment and partisan politics, unhappy with personal or family attacks, and disgruntled with the behavior of constituents.

Finally, we turn to the unaffected group–those for whom school board service had no effect on their desire to run for higher office. Descriptive statistics indicate that relative to the other two groups, this population is more likely to be older. They are less likely to have children at home or to be employed, with a substantial portion being retired. They are more likely to be embedded in small towns and rural areas, which might have offered a buffer against the partisan divisions in the cities. Respondents in the unaffected group are less likely to be members of political groups, but they have much higher rates of previously serving in elected positions.

While overall we do find fairly clear constellations of these groups across our analyses, we are limited in that we are relying on self-reported survey data and qualitative comments. It is possible that those planning on exiting politics are more inclined to view their school board experience negatively on reflection to justify their decision. Conversely, those planning on staying or expanding their political ambitions may reflect more positively on their experiences. Thus, we recognize that a limitation of our data is that the experiences, beliefs and expectations of participants could influence their responses in a variety of ways. Finally, the focus of the study emphasizes democratic elections and processes; school board governance will be different in cases where school board members are appointed or in situations where educational decisions and messages are tightly controlled by the national government.

We find that the majority of our respondents state that it is unlikely that they will run for higher office. For those who express ambition, the greatest interests are for local or state political office. As expected, political ambition is higher for those who have stated that serving on the school board made it more likely they would run for higher office. This group also has grander ambitions, with about a quarter interested in the U.S. House or U.S. Senate.

## Conclusions and directions for future research

The headlines of the past several years suggest that school board trustees are serving in tumultuous times, perhaps driven not to run for reelection or to seek higher office. The story we tell is more complex, and more interesting. We find that this is true in non-rural districts among trustees who describe their experience in terms of toxicity, hyper-politicization, and dirty politics.

In these same districts, though, are trustees who became energized by their service and are considering running for higher office. They are more likely to be Democrats, and those who are not overly concerned about the personal and professional tolls a future election might have on them. They like their work and get satisfaction from serving. Some are excited to be a voice for populations within their district who they feel are underrepresented.

Our research also suggests there is more work to be done understanding the diversity of types of districts. As one of our respondents reminds us, rural districts are very different than their urban and suburban counterparts. In some of these very small districts where people are known to one another, serving in elected positions harkens back to a time of shared civic duty. In gathering our data, we even saw members of the same family serving together on school boards in small and rural districts. Additionally, these districts tend to be more homogenous on demographic characteristics, which could possibly lead to less differences in opinion over issues of COVID protocols and school curricula on race, sex, and other social issues.

This also points to a limitation of our study: not all school boards are politically contentious. We chose our case study of Texas in part because there had been documented cases of school board meetings marked by polarization and rancorous proceedings. However, at the same time, Texas is a large state with diversity in its civic life and school board experiences, and unsurprisingly some respondents did not view themselves to be housed in contentious districts. Because we do not directly measure the internal dynamics of school boards, and rely on self-evaluations which can be biased, we are not able to fully capture the effect of the political atmosphere on political ambition. At the same time, based on what we do find in the respondents’ comments, this is a potentially promising area of research. Future studies could better measure and model the effects of partisanship and political conflict on ambition to flesh out and map the effects of such factors on the decision to continue in school board service and politics more generally.

While not all of respondents were affected by the politics and pandemic of the past few years, it is also fair to say that for some it made quite a difference in encouraging, or discouraging, their school board work or plans for future public office. If this current hyper focus on school boards as a political and social battleground continues, it will be an important task for researchers to continue to understand the effects of such attention, particularly as it inspires some individuals and dissuades others. This could have a lasting impact on the types of candidates that enter, and stay, in the political arena, for school board service and beyond.
